# Telehealth for Nutritional Care: A Tool for Improving Patient Flow in Hospitals

**DOI:** 10.1089/tmr.2021.0054

**Published:** 2022-06-28

**Authors:** Mayumi Shima, Silvia Maria Fraga Piovacari, Milton Steinman, Andrea Z. Pereira, Oscar Fernando Pavão dos Santos

**Affiliations:** ^1^ Clinical Nutrition Service, Hospital Israelita Albert Einstein, São Paulo, Brazil.; ^2^ Supervisor of the General Surgery Residency and Professor of Medicine, Emergency Surgery, Hospital Israelita Albert Einstein, São Paulo, Brazil.; ^3^ Oncology and Hematology Department, Hospital Israelita Albert Einstein, São Paulo, Brazil.; ^4^ Associate Professor of Medicine, Nephrology, Hospital Israelita Albert Einstein, São Paulo, Brazil.

**Keywords:** telehealth, telemedicine, dietary counseling, patient flow

## Abstract

**Background:**

Hospitals are constantly searching for opportunities to improve efficiency, and telehealth (TH) has recently emerged as a strategy to assist in patient flow. We evaluated two methods of dietary counseling offered to patients in the time period between the medical and final hospital discharge. Counseling was given either *via* the TH group or the face to face (FTF) group to the patients and their respective impact was evaluated on the patients' satisfaction and on the hospital patient flow.

**Methods:**

This study was a prospective, randomized clinical trial where patients were randomized to receive dietary counseling *via* TH (use of tablet) or FTF at the time of hospital discharge. We evaluate the duration of time between medical discharge and hospital discharge; between requesting dietary counseling and dietitian's arrival; and duration of dietary counseling. At the end of dietary counseling, both groups received a patient satisfaction questionnaire to answer.

**Results:**

A total of 159 patients were randomized to receive dietary counseling *via* TH (TH, *n* = 78) or FTF (FTF, *n* = 81). The two groups TH and FTF did not differ in terms of the median time between (1) medical and hospital discharge; (2) requesting counseling and the dietitian's arrival; and (3) duration of dietary counseling. Both groups mostly reported being “satisfied” or “above expectations,” and the FTF group scored “highest satisfaction” more often relative to the dietitian's work and interaction and on confidence in the dietitian's orientations. Finally, in the TH group, 90.7% graded likely-4 or very likely-5 when asked whether dietary counseling can be conducted entirely *via* TH, and 92% answered “4” or “5” when asked whether they would recommend dietary counseling *via* TH.

**Conclusions:**

Although the FTF group had a greater overall satisfaction relative to the TH group, TH proved to be a useful tool for dietary counseling.

The trial has only Institutional Review Board approval (protocol 2685-16).

## Introduction

Hospitals worldwide are searching for ways to improve efficiency. A major challenge is to manage the rising demands without an equivalent budget increase while maintaining or even improving standards of quality and access. Reducing length of stay can generate a “virtual capacity” equivalent to building at least one additional ward at a fraction of the cost.^1^ Poor patient flow impairs patient and staff satisfaction, as well as effective utilization of resources.^2^

Planning for hospital discharge is a time-consuming activity involving nutritional counseling, medication reconciliation, scheduling a return medical appointment, and arranging for adequate transportation.^1,3^ Among the various stages of patient flow, the discharge process could be significantly improved.

To this end, telehealth (TH) may become a valuable strategy. TH seems particularly appropriate in the current worldwide context of the aging population and higher prevalence of chronic diseases, and the mounting pressure to control public and private spending.^4,5^ Studies have reported the successful use of TH for clinical nutrition in remote or rural areas,^6–8^ however, there is a great potential for its use also in urban areas and where there is no specialist for dietary counseling. As TH care services gain widespread acceptance by both patients and health professionals, new opportunities are developing for the registered dietitian nutritionist.^9^

Thus, the aim of this study was to evaluate the impact of dietary counseling *via* TH in comparison with the traditional method on the duration of the time interval between medical discharge and hospital discharge as well as on the degree of patient satisfaction.

We hypothesized that the use of TH would result in a decrease of time between requesting dietary counseling and dietitian's arrival, and that the duration of dietary counseling in both groups will not show a significant difference. Furthermore, we hypothesized that satisfaction in both groups would be similar. The primary objective of our study was to compare time measurements between participants receiving TH and traditional method, if this would improve patient flow. And the satisfaction evaluation was defined as a secondary outcome.

## Materials and Methods

### Study design and patients

This was a prospective, randomized clinical trial conducted between March 2016 and August 2017 in one private hospital (in São Paulo, Brazil). The Institutional Review Board approved the study (protocol 2685-16). All participants received and signed an informed consent form before participation and anonymity of the patients was guaranteed. Patients from all units of hospital the doctor prescribed dietary counseling, male or female subjects, aged >18 years, receiving an oral diet and none having difficulty in understanding instructions were included.

### Randomization

Patients were randomly assigned in a 1:1 ratio to the intervention (TH group) or control arms (face to face [FTF] group). The randomization list was computer-generated and balanced by blocks of undisclosed size. Patients were informed of their allocation before the hospital discharge and dietary counseling.

### Intervention

Patients were randomized to receive dietary counseling *via* the TH–TH group- (TH group) or FTF–FTF group- (FTF group) at the time of hospital discharge. Patients from both groups received a written dietary prescription to be followed at home.

The patients of the TH group received a 16GB Ipad Air tablet^*®*^ with the installed *Vsee^®^* program to give them real-time dietary counseling, based on the individual's dietary prescription. The encounters were conducted with real-time audio and video interaction between the patient and the dietitian. The patients received help from a dietitian to use the tablet.

In both groups, counseling was done by explaining the dietary prescription and answering potential questions from the patients.

### Data collection

All data were obtained by trained dietitians through the use of standard structured questionnaires. Information on age, gender, clinical diagnosis and procedures, comorbidities, dietary prescription, nutritional care level, and medical and hospital discharge times was obtained from the electronic medical records.

“Medical discharge” was defined as the time the medical doctor prescribes the discharge and “hospital discharge” as the time the patient actually leaves the hospital after receiving all of the medical team's orientations.

We also recorded the time at which the nurse requested dietary counseling and the duration of the dietitian's orientation. Actual time was noted to calculate duration between medical discharge and hospital discharge; between requesting dietary counseling and dietitian's arrival; and duration of dietary counseling. Data were collected between December 2016 and May 2017.

### Satisfaction questionnaire

Following dietary counseling, both groups received a patient satisfaction questionnaire adapted from a previously tool used by the telemedicine of the hospital, using a five-level Likert scale. We collected some information about the subjective opinion. Questions 4 and 5 were given only to the TH group.

Questions:

(1) How satisfied are you with the dietary counseling you received from the dietitian?
(2) How did you feel about your interaction with the dietitian?
(3) How confident do you feel about the dietary orientations you received?
(4) Do you think dietary counseling can be conducted entirely *via* TH?
(5) Would you recommend dietary services *via* TH to other people?

The answers to questions 1–3 were as follows: 1 = very dissatisfied, 2 = dissatisfied, 3 = satisfied, 4 = very satisfied, and 5 = above expectations; for questions 4 and 5, the answers were as follows: 1 = never, 2 = most likely, 3 = perhaps, 4 = likely, and 5 = very likely.

The questionnaire was applied on the article after dietary counseling; the completion was not confidential, but anonymity of the patients was guaranteed.

### Statistical analyses

The sample size was estimated using data collected in 2015 from the Clinical Nutrition Service of hospital, taking into account the time period from the moment that dietary counseling was requested by the attending nurse to the moment the patient received the service, as well as the total duration of dietary counseling, in holidays and weekends. The tests were performed at the 5% significance level and power at 90%, and the required number of subjects was estimated to be 100 per arm.

Data were expressed by median and interquartile range (1°, 3° quartiles) in case of quantitative variables and absolute frequencies and percentages for the qualitative variables. Frequency distribution was analyzed using boxplots and histograms.

Mann–Whitney tests were used to compare numerical variables, and chi-square or Fisher's exact tests were used for categorical measurements. The total time of dietary counseling was compared between groups using general linear models adjusted for gamma distributions and logarithmic range functions. Next, to determine which patient and care variables should be considered to make the adjustments, we used a stepwise model and the Akaike information criterion to include only variables with *p*-value <0.05. The results of the adjusted models were expressed by the estimated effects of the mean time of dietary counseling, obtained by the exponential value of the estimated coefficients and the *p*-value. Statistical analyses were conducted using the R 3.1.3 software.

## Results

### Participants

**FIG. 1.**
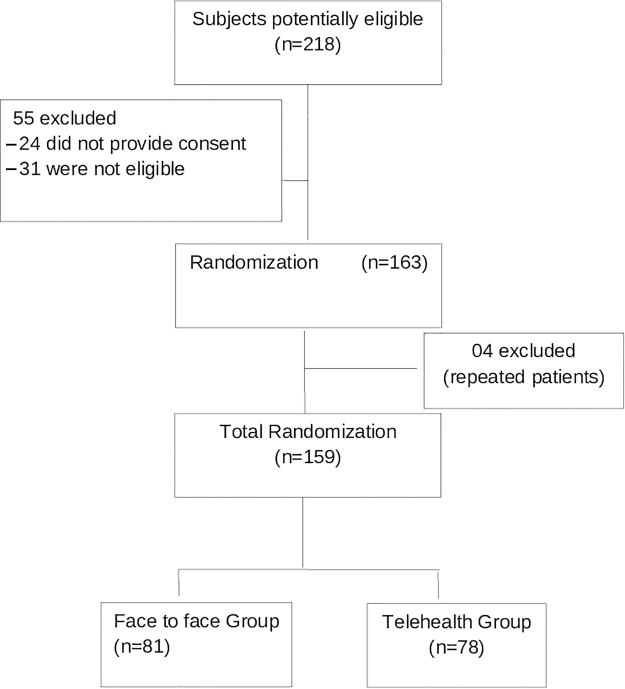
Flowchart of the study.

Figure 1 shows the flowchart of participants' inclusion. During the study, 218 patients were eligible, but 55 patients were excluded (24 did not provide consent to participate and 31 subjects who eventually did not meet all the criteria), and 163 subjects were enrolled and randomized. At the end, the study consisted of 159 patients (78 patients were randomized to the TH group and 81 patients to the FTF group), 4 patients were excluded because they were randomized twice, in different admissions.

### Patient characteristics

Baseline characteristics were similar between the two arms. The majority of participants were females (51.6%), and the median age was 53 years (range 18–95 years). We did not find any significant difference between the TH and FTF groups in terms of age, gender, diagnosis, comorbidities, or submission to surgery.

The most prevalent comorbidity among all patients was hypertension (FTF *n* = 25, 30.9%; TH *n* = 24, 30.8% *p* < 0.99), followed by dyslipidemia (FTF *n* = 12, 14.8%; TH *n* = 12, 15.4% *p* < 0.99). At the time of admission, the primary diagnosis of 144 patients (FTF *n* = 77, 95.1%; TH *n* = 67, 85.9% *p* < 0.08) was gastrointestinal, and 83 patients (FTF *n* = 43, 53.1%; TH *n* = 40, 51.3% *p* < 0.94) underwent surgery (Table 1).

**Table 1.** Patients' Profile Undergoing Dietary Counseling *via* Telehealth or *via* Presential Face to Face Interaction with the Dietitian

Characteristics	FTF (*n* = 81), *n* (%)	TH (*n* = 78), *n* (%)	*p*
Median age (years) [IQR]	57.00 [42.00; 69.00]	48.00 [38.00; 65.75]	0.18
Sex			
Female	40 (49.4)	42 (53.8)	0.68
Male	41 (50.6)	36 (46.2)	
Dyslipidemia			
No	69 (85.2)	66 (84.6)	>0.99
Yes	12 (14.8)	12 (15.4)	
Hypothyroidism			
No	74 (91.4)	72 (92.3)	>0.99
Yes	7 (8.6)	6 (7.7)	
Hypertension			
No	56 (69.1)	54 (69.2)	>0.99
Yes	25 (30.9)	24 (30.8)	
Diabetes mellitus			
No	74 (91.4)	67 (85.9)	0.40
Yes	7 (8.6)	11 (14.1)	
Gastrointestinal primary diagnosis			
No	4 (4.9)	11 (14.1)	0.08
Yes	77 (95.1)	67 (85.9)	
Surgery			
No	38 (46.9)	38 (48.7)	0.94
Yes	43 (53.1)	40 (51.3)	

*p*-Values by Mann–Whitney testing to numerical variables and chi-square testing to categorical variables.

FTF, face to face; IQR, interquartile range (1°; 3° quartiles); TH, telehealth.

We included patients from all units of the hospital, but most came from the gastroenterology service, which had the highest rate of dietary orientation requests. The most frequent pathologies were gastroesophageal reflux, gastroenteritis, diverticulitis, diverticular disease, abdominal pain, and appendicitis; and surgeries were gastroesophageal reflux correction, retosigmoidectomy, appendectomy, cholecystectomy, and gastroplasty.

### Dietary counseling

In terms of dietary counseling, the only factor that differed between groups was that dietary counseling occurred mostly on the weekends or holidays for the TH group (*p* = 0.005; Table 2).

**Table 2.** Dietary Counseling Profile of Patients Undergoing Dietary Counseling *via* Telehealth or *via* Presential Face to Face Interaction with the Dietitian

Variables	FTF (*n* = 81)	TH (*n* = 78)	*p*
	*n* (%)	*n* (%)	
Weekends and holidays			
No	47 (58.0)	27 (34.6)	0.005
Yes	34 (42.0)	51 (65.4)	
Nutritional care level			
No classification	8 (9.9)	8 (10.3)	0.54
Primary	11 (13.6)	8 (10.3)	
Secondary	56 (69.1)	51 (65.4)	
Tertiary	6 (7.4)	11 (14.1)	
Consistency of diet			
Liquid	5 (6.2)	6 (7.7)	0.94
Creamy	23 (28.4)	14 (17.9)	0.17
Pureed	9 (11.1)	12 (15.4)	0.57
Soft	25 (30.9)	23 (29.5)	0.98
Mechanical soft	13 (16.0)	16 (20.5)	0.60
Regular	10 (12.3)	9 (11.5)	>0.99
Nutritional supplements			
No	75 (92.6)	70 (89.7)	0.72
Yes	6 (7.4)	8 (10.3)	
Dietary modifications			
Low in fat	7 (8.6)	2 (2.6)	0.16
Low sodium	5 (6.2)	4 (5.1)	>0.99
High in fiber	3 (3.7)	3 (3.8)	>0.99
Low fiber	17 (21.0)	28 (35.9)	0.05
Diabetic	11 (13.6)	11 (14.1)	>0.99
Lactose free	11 (13.6)	5 (6.4)	0.21
Others	6 (7.4)	8 (10.3)	0.72
No restrictions	32 (39.5)	27 (34.6)	0.63
Number of restrictions			
0	32 (39.5)	27 (34.6)	0.49
1	41 (50.6)	41 (52.6)	
2	5 (6.2)	10 (12.8)	
3	3 (3.7)	0 (0.0)	

*p*-Values by Mann–Whitney testing to numerical variables and chi-square or Fisher exact testing to categorical variables.

The majority of patients were classified secondary nutritional care level (67.3%), and the most diet consistency oriented was soft (30.2%). When dietary modification was prescribed, the most prevalent was the low-fiber diet (28.3%), followed by a diabetic diet (13.8%) and a lactose-free diet (10.1%).

### Analysis of time measurements

Analysis of duration between medical discharge and hospital discharge; between requesting dietary counseling and dietitian's arrival; and duration of dietary counseling was calculated in minutes. There was no significant difference between the two groups in terms of the following: median time between medical and hospital discharge (FTF: 112 min; TH: 116.5 min; *p* < 0.43), median time between requesting dietary counseling (from the attending nurse) and the dietitian's arrival (FTF: 33 min; TH: 36.5 min; *p* < 0.17), or the median total time of dietary counseling (FTF: 15 min; TH: 13.5 min; *p* < 0.052) (Table 3).

**Table 3.** Median Times (Minutes) Patients Undergoing Dietary Counseling *via* Telehealth or *via* Presential Face to Face Interaction with the Dietitian

Duration of outcome measured time periods in minutes	FTF (*n* = 81)	TH (*n* = 78)	*p*
	Median [1°; 3° quartiles]	Median [1°; 3° quartiles]	
Between medical discharge and hospital discharge	112.00 [63.00; 161.00]	116.50 [74.00;172.50]	0.43
Between requesting dietary counseling (from the nurse) and dietitian's arrival	33.00 [16.00; 54.00]	36.50 [22.00; 63.00]	0.17
Duration of dietary counseling	15.00 [12.00; 20.00]	13.50 [10.00; 18.00]	0.052

*p*-Values by Mann–Whitney testing.

Although no significant differences were detected, we further compared the groups using a multiple adjustment model controlling for all other variables. Table 4 shows that the dietary orientation time was 16% (2 min, 24 sec) shorter for the TH group (*p* = 0.03). This result was controlled for patient and nutritional care variables, including care on weekends or holidays, which was associated with an 18% mean increase in dietary counseling time (*p* = 0.04), and the patient's gender, with 16% shorter orientation times for males (*p* = 0.02).

**Table 4.** Adjusted Effect of the Counseling Group on the Dietary Counseling Time

Factors	Adjusted effect	*p*
Counseling group		
Face to face (reference)	1.00	0.03
Telehealth	0.84	
Weekend/holidays vs. Monday to Friday		
Monday to Friday (reference)	1.00	0.04
Weekends/holidays	1.18	
Sex		
Female (reference)	1.00	0.02
Male	0.84	

Estimates and *p*-values obtained by Gamma models.

When the patient profile and dietary counseling profile were analyzed separately, no significant effects were observed for the mean orientation time (data not shown).

### Satisfaction questionnaire

Table 5 shows that, overall, patients reported being satisfied with the care or considered it being above their expectations. However, when we compared groups, we found that relative to the TH group, the FTF group had a greater number of “above expectations” responses in their satisfaction with the dietitian's orientation (*p* = 0.004), in how they felt about their contact with the dietitian (*p* = 0.003), and also in terms of how confident they felt about the orientation (*p* = 0.013).

**Table 5.** Satisfaction with Dietary Counseling of Patients Undergoing Dietary Counseling *via* Telehealth or *via* Presential Face to Face Interaction with the Dietitian

Satisfaction	FTF (*n* = 81)	TH (*n* = 78)	*p*
	*n* (%)	*n* (%)	
How satisfied are you with the dietary counseling you received from the dietitian?			
Very dissatisfied	None	None	
Dissatisfied	None	None	
Satisfied	2 (2.5)	8 (11.4)	0.004
Very satisfied	13 (16.0)	19 (27.1)	
Above expectations	66 (81.5)	43 (61.4)	
No response	None	8	
How did you feel about your interaction with the dietitian?			
Very dissatisfied	None	None	
Dissatisfied	None	None	
Satisfied	1 (1.2)	8 (11.4)	0.003
Very satisfied	11 (13.6)	16 (22.9)	
Above expectations	69 (85.2)	46 (65.7)	
No response	None	8	
How confident do you feel about the dietary orientation you received?			
Very dissatisfied	None	None	
Dissatisfied	None	None	
Satisfied	1 (1.2)	10 (14.3)	0.01
Very satisfied	13 (16.0)	13 (18.6)	
Above expectations	67 (82.7)	47 (67.1)	
No response	None	8	
Number of patients who commented on the counseling received			
No comment	71 (87.7)	71 (91.0)	0.66
Comment	10 (12.3)	7 (9.0)	

*p*-Values obtained by Mann–Whitney testing to numerical variables and chi-square testing to categorical variables.

Next, the TH group was questioned separately regarding their satisfaction with TH. Three patients in this group did not respond. Of the 75 patients who responded to the two questions, 90.7% (*n* = 68) responded *4* (likely) or *5* (very likely) when asked whether dietary counseling can be conducted entirely *via* TH, and 92% (*n* = 69) responded *4* (likely) or *5* (very likely) when asked whether they would recommend dietary counseling *via* TH (Table 6).

**Table 6.** Evaluation of Telehealth for Dietary Counseling by the Recipient Patients

TH group	*n* (%)
Do you think dietary counseling can be conducted entirely *via* TH?	
1. Never	Zero
2. Most likely	2 (2.7)
3. Perhaps	5 (6.7)
4. Likely	18 (24.0)
5. Very likely	50 (66.7)
No response	3
Would you recommend dietary services *via* TH to other people?	
1. Never	Zero
2. Most likely	Zero
3. Perhaps	6 (8.0)
4. Likely	14 (18.7)
5. Very likely	55 (73.3)
No response	3
Number of patients who commented on the counseling received	
No comment	59 (75.6)
Comment	19 (24.4)

## Discussion

The TH group did not differ significantly from the FTF group in terms of time between medical and hospital discharge, time between request for orientation and the dietitian's arrival, or in the total time of dietary counseling. However, a second multiple model analysis revealed that the duration of dietary counseling was in fact 16% (2 min, 24 sec) shorter for the TH group (*p* = 0.03). Furthermore, orientation performed on weekends or holidays was significantly longer (*p* = 0.04), and dietary counseling time was shorter for male patients (*p* = 0.02).

Overall, patients in both groups reported being satisfied with the care or considered it being above their expectations, and no one responded “dissatisfied” or “very dissatisfied” to any of the questions. However, when we compared groups, we found that the FTF group had a relatively greater number of “above expectations” responses in their satisfaction with the dietitian's orientations (*p* = 0.004), in how they felt about their contact with the dietitian, (*p* = 0.003), and in terms of how confident they felt about the orientation (*p* = 0.01).

For questions asked only of the TH group, 90.7% (*n* = 68) responded 4 (likely) or 5 (very likely) when asked whether dietary counseling can be conducted entirely *via* TH, and 92% (*n* = 69) responded 4 (likely) or 5 (very likely) when asked whether they would recommend nutritional care *via* TH.

Other studies evaluating TH have also shown good satisfaction, including videoconferencing of home parenteral nutrition care,^10,11^ nutritional screening in cancer patients,^12^ video teleconsultation in a virtual diabetes clinic,^13^ cancer support groups for American Indian and Alaska Native patients,^14^ virtual obstetric care,^15^ virtual visits for the follow-up of chronic conditions,^16^ and telemedicine for heart failure patients.^17^ The use of TH for nutritional counseling is recent in Brazil, which may explain why satisfaction with FTF remains higher than that with the TH group. However, the fact that ratings for TH were high in our group suggests its great potential use for dietary counseling during the interval between medical and hospital discharge.

In a study that assessed the perception of teleconsultation, the patients reported as positive points the quality of care, convenience, cost reduction (e.g., with transportation), and punctuality of the consultations. However, some patients also reported negative points, such as feeling a sense of alienation and diminished doctor/patient rapport, suggesting poorer clinical care. Furthermore, while some TH patients cited practicality and innovation as positive points (and said they would recommend it), others suggested that FTF counseling should not be replaced and expressed concern for the treatment of elderly patients. In conclusion, all these aspects and opinions should be considered when planning new services.^18^

Here, we evaluated the effect of TH on patient flow by comparing dietary counseling time and patient satisfaction between groups receiving dietary counseling *via* TH *versus* FTF counseling in the time period between the time of medical and final hospital discharge.

This study had some limitations, namely: patients and dietitians were not blind to group assignments, the satisfaction questionnaire used was not validated and it was given following the dietary counseling (which could be biased by the presence of the dietitian), the fact that we did not assess retention of information following counseling, and the number of subjects was minor than estimated. Nevertheless, all participant dietitians applied the nutrition unit's standard practice guidelines for dietary counseling and were familiar with both FTF and TH groups.

Although the differences in counseling duration between groups revealed by the multiple model analysis were small, TH could offer some time advantage for hospitalized patients in situations when all dietitians are on call and busy (i.e., TH care could be conducted by a dietitian assigned to the call center). In this case, the TH could contribute to improve patient flow. TH could also be used for home follow-up of patients after hospital discharge,^19^ allowing quick and easy answers to common questions and assessing adherence to the nutritional guidelines, especially for patients with limited mobility. In addition, including TH into dietetic practice can enhance the efficiency and quality of nutrition care and counseling delivered by dietitians.^20,21^

Previous studies have reported the successful use of TH for clinical nutrition in remote or rural areas.^6–8,22–25^ As mentioned above, while our study took place in an urban setting, TH could be particularly useful when the number of available professionals is limited^26,27^ or where transportation within a large metropolitan area is difficult. Increased use of TH has been seen during the current COVID-19 health crisis. Currently, TH visits also offer the ability to keep patients and health care providers safe, and allow for the continued care of patients.^28^

## Conclusions

Although the FTF group had a greater overall satisfaction relative to the TH group, TH proved to be a useful tool for dietary counseling. There is a growing interest in the use of TH and future studies should continue to test its efficiency and ideal applicability.

## Abbreviations Used

FTF: face to face
IQR: interquartile range
TH: telehealth
